# Comprehensive Analysis of Cuproptosis Genes and Identification of Cuproptosis Subtypes in Breast Cancer

**DOI:** 10.2174/1386207326666230120112904

**Published:** 2023-04-04

**Authors:** Jialin Li, Lei Li, Yi Dong, Bin Zhong, Wei Yin

**Affiliations:** 1 Clinical College, Wuhan University of Science and Technology, Wuhan 430000, China;; 2 Tianyou Hospital of Wuhan University of Science and Technology, Wuhan 430000, China;; 3 General Hospital of Central Theater Command, Wuhan 430000, China;; 4 Southern Medical College University, University, Guangzhou 510000, China

**Keywords:** Cuproptosis, BRCA, subtypes, ICI treatment, cell death, copper-induced death

## Abstract

**Background:**

Copper-induced death (cuproptosis) is copper-dependent regulated cell death, which is different from known death mechanisms and is dependent on mitochondrial respiration. However, its effect on breast cancer (BRCA) is unclear.

**Objective:**

The objective of this study is to explore the important clinical significance of cuproptosis genes and to provide a new idea for guiding the personalized immunotherapy strategy of BRCA patients.

**Materials and Methods:**

We collected cuproptosis genes from published work. The gene alteration, differential expression, and prognostic value of cuproptosis genes were explored in BRCA based on TCGA database. We identified two subtypes (clusters A and B) by performing unsupervised clustering. The difference between two clusters was deeply explored, including clinical features, differential expressed genes (DEGs), pathways, and immune cell infiltration. Based on the DEGs between two clusters, a cuproptosis score was constructed and its predictive capability for overall survival of BRCA patients was validated.

**Results and Discussion:**

Patients with high cuproptosis score have worse survival status, with an increased infiltration level of most immune cells. Further analysis suggested that BRCA patients with high cuproptosis score may be sensitive to immune checkpoint inhibitor (ICI) treatment.

**Conclusion:**

Our findings may improve our understanding of cuproptosis in BRCA and may distinguish patients suitable for ICI treatment.

## 1 INTRODUCTION

Copper-induced death (Cuproptosis) is a new cell death mechanism [1]. Cuproptosis is distinct from all other known mechanisms of regulated cell death, including apoptosis, ferroptosis, pyroptosis, and necroptosis [2]. Seven genes, including LIAS, LIPT1, FDX1, DLD, DLAT, PDHA1, and PDHB, were proven to induce cuproptosis progress. However, the role of cuproptosis in breast cancer (BRCA) was not fully understood. Thus, more research should be done to reveal the role and mechanism of cuproptosis in tumor progression of BRCA. In our study, we comprehensively evaluated the expression, genetic alteration, and prognosis of cuproptosis genes in BRCA. Further, based on the expression of cuproptosis genes, we performed unsupervised clustering and identified two subtypes (clusters A and B) of BRCA patients. Interestingly, the survival status of BRCA patients in two clusters was significantly different, indicating that a potential cuproptosis-related mechanism need to be explored. Thus, we further explored the difference between two clusters, including clinical features, differential expressed genes (DEGs), pathways, and immune cell infiltration. We also constructed the cuproptosis score, which could predict the overall survival of BRCA patients. The correlation between cuproptosis score and tumor immune microenvironment and immunotherapeutic efficacy was further explored.

## 2 METHODS AND MATERIALS

### 2.1 Data Collection

The mRNA expression profile and clinical data of TCGA-BRCA (N = 1097) were downloaded from UCSC-Xena database (https://xenabrowser.net/datapages/). The GSE20685 (N = 327) was obtained from Gene Expression Omnibus (GEO) database (https://www.ncbi.nlm.nih.gov/geo/). R package “limma” and “sva” were used to eliminate batch effect. The gene set of cuproptosis was downloaded from published work [2], with 7 genes enrolled LIAS, LIPT1, FDX1, DLD, DLAT, PDHA1, and PDHB).

### 2.2 Online Analysis

(1)The genetic alteration of cuproptosis genes, including copy number variant (CNV) and mutation, were conducted using GSCA database (http://bioinfo.life.hust.edu.cn/GSCA/#/). (2) The immunophenoscore (IPS) score of BRCA patients, which could predicted the therapeutic effect of immune checkpoint inhibitor (ICI) treatment, were obtained from TCIA database (https://tcia.at/home). (3) TISCH database (http://tisch.comp-genomics.org/) was used to explore the expression of cuproptosis genes in single cell data of BRCA.

### 2.3 Enrichment Analysis

The pathways used in Gene Set Variation Analysis (GSVA) were downloaded from Msigdb database, including HALLMARY, Reactome pathways and Kyoto Encyclopedia of Genes and Genomes (KEGG) pathways. R package “GSVA” was used to calculate the pathway score of each sample based on the TCGA-BRCA and GSE20685. The R package “clusterprofiler” was used to conduct Gene Ontology (GO) and KEGG analysis of DEGs.

### 2.4 Immune Cell Infiltration Analysis

The R package “ESTIMATE” was used to assess the tumor microenvironment of BRCA samples, including stromalscore, immunescore, and ESTIMATEscore (stromalscore plus immunescore). The ssGSEA function of R package “GSVA” was used to assess the infiltration level of 23 immune cells. The immune cell level was further compared in variant groups.

### 2.5 Construction of Cuproptosis Score

The 174 DEGs between two clusters were identified using R package “limma” by |logFC| > 1, *P <* 0.05. Further univariate regression analysis identified 27 DEGs related to overall survival (*p <* 0.001). We then curated the final 27 DEGs determined to conduct principal component analysis (PCA), and principal components 1 and 2 were extracted to construct the cuproptosis score [3].

### 2.6 Statistical Analysis

**Fig. (1):**
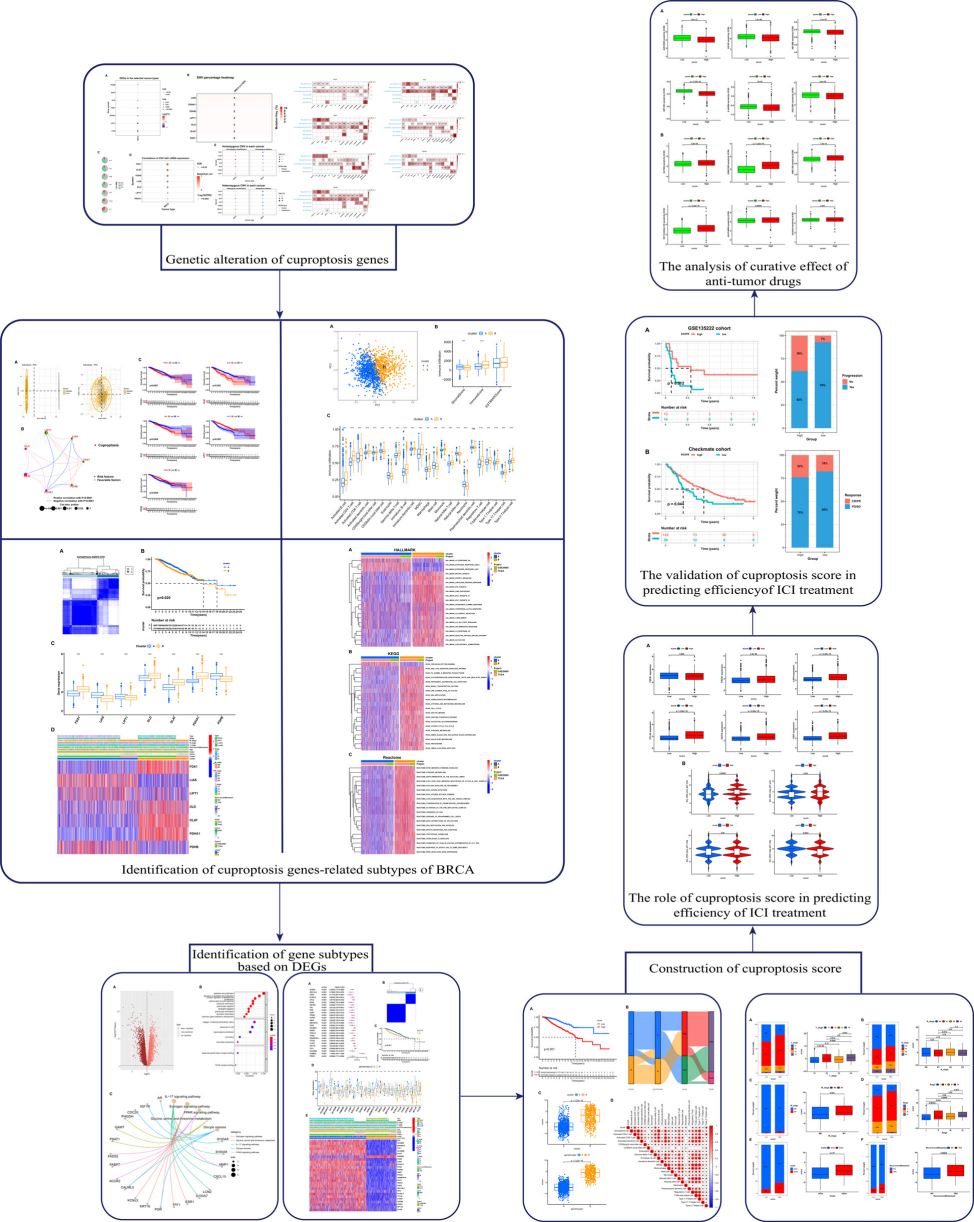
Workflow diagram. The specific workflow graph of data.

Data are presented as the mean ± SD. Differences between the groups were analyzed using Student’s t-test. Statistical analysis was performed using R 4.1.1 *P <* 0.05 (two-tailed) was considered statistically significant: *p < 0.05, **p < 0.01, ***p < 0.001, and ****p < 0.0001. The overall flow diagram is shown in (Fig. **1**).

## 3 RESULTS

### 3.1 Genetic Alteration of Cuproptosis Genes

**Fig. (2):**
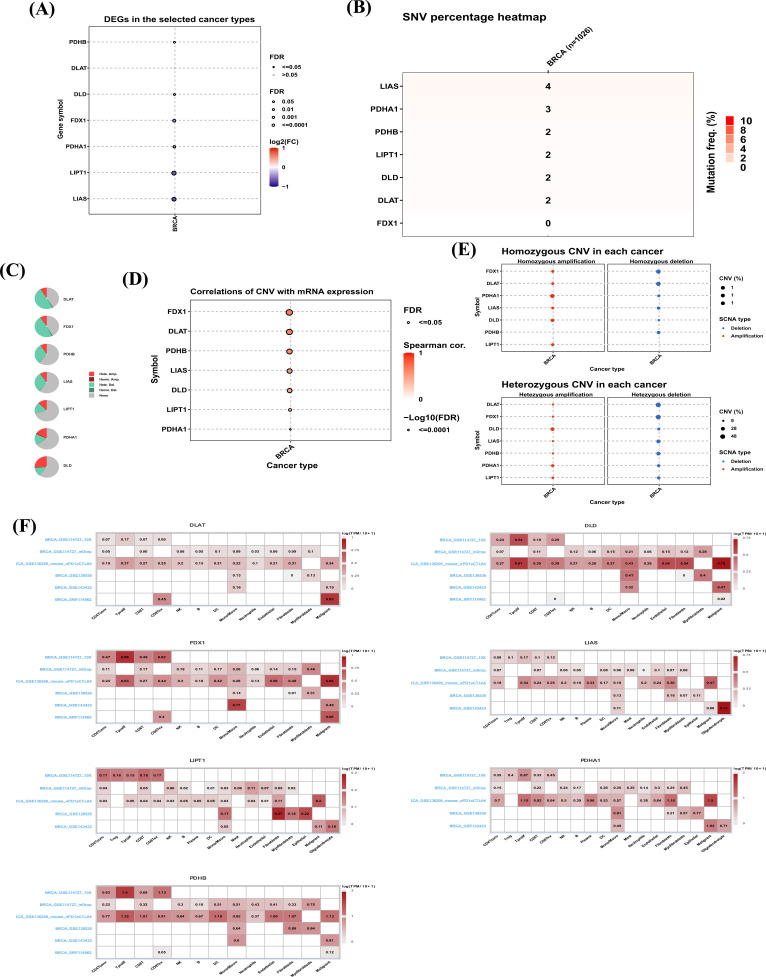
(**A**) Comprehensive analysis of cuproptosis genes. The differential expression of cuproptosis genes in BRCA. (**B**) The frequency of deleterious mutations of cuproptosis genes in BRCA. (**C**) The CNV percentage of cuproptosis genes in BRCA. (**D**) The correlation of CNV with mRNA expression in BRCA. (**E**) The homozygous and heterozygous CNV of cuproptosis genes in BRCA. (**F**) The single cell expression of cuproptosis genes in BRCA.

Our study included a total of 7 cuproptosis genes. We first explored the differential expression of cuproptosis gene in normal and tumor tissues in BRCA. Results indicated that PDHB was highly expressed in BRCA, while DLD, FDX1, PDHA1, LIPT1, and LIAS were lowly expressed in BRCA (Fig. **2A**). In addition, the mutation frequency of cuproptosis genes was low in BRCA (Fig. **2B**). CNV of 7 cuproptosis genes was further assessed. Among them, DLD has the highest amplification frequency in BRCA (Fig. **2C**). The mRNA expression of all 7 genes was positively correlated with their CNV levels (Fig. **2D**). We also represented the percentage of homozygous CNV and heterozygous CNV of each gene in BRCA, including homozygous amplification, homozygous deletion, heterozygous amplification, and heterozygous deletion (Fig. **2E**). We also explored the single cell expression of 7 cuproptosis genes in BRCA using TISCH database. All these genes were generally expressed in tumor microenvironment (Fig. **2F**).

### 3.2 Identification of Cuproptosis Genes-related Subtypes of BRCA

**Fig. (3):**
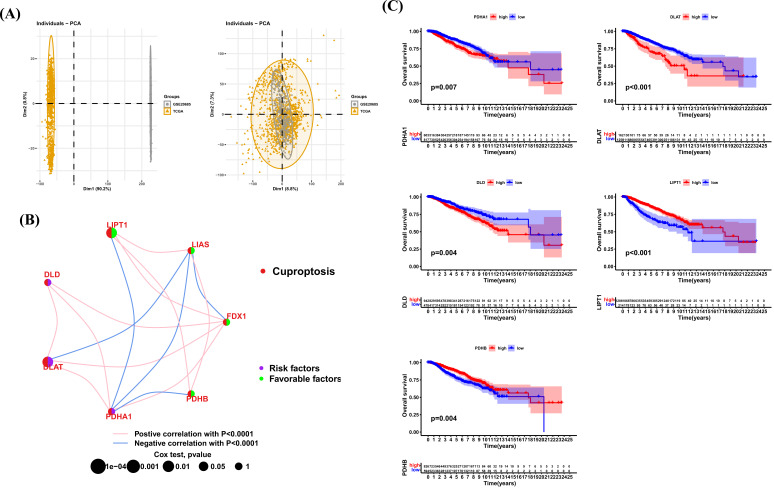
Correlation and prognostic analysis of cuproptosis genes in BRCA. (**A**) The PCA plot of BRCA samples before and after data merge. (**B**) Correlation and prognostic value of cuproptosis genes in BRCA. The line connecting the cuproptosis genes represents their correlation, with the line thickness indicating the strength of the correlation between cuproptosis genes. Blue and pink represent negative and positive correlations, respectively. (**C**) Kaplan-Meier analysis of indicated cuproptosis genes in BRCA.

We first merged the TCGA-BRCA and GSE20685 data (Fig. **3A**). By performing correlation and univariate regression analysis, we described the comprehensive landscape of cuproptosis genes, including their correlation and prognostic value in BRCA (Fig. **3B**). For example, PDHA1 and DLAT had a positive correlation and were both risk factors in BRCA. Kaplan-Meier analysis indicated that BRCA patients have worse overall survival with high expression of PDHA1, DLAT, and DLD (Fig. **3C**). On the contrary, BRCA patients with high expression of LIPT1 and PDHB have better survival status.

**Fig. (4):**
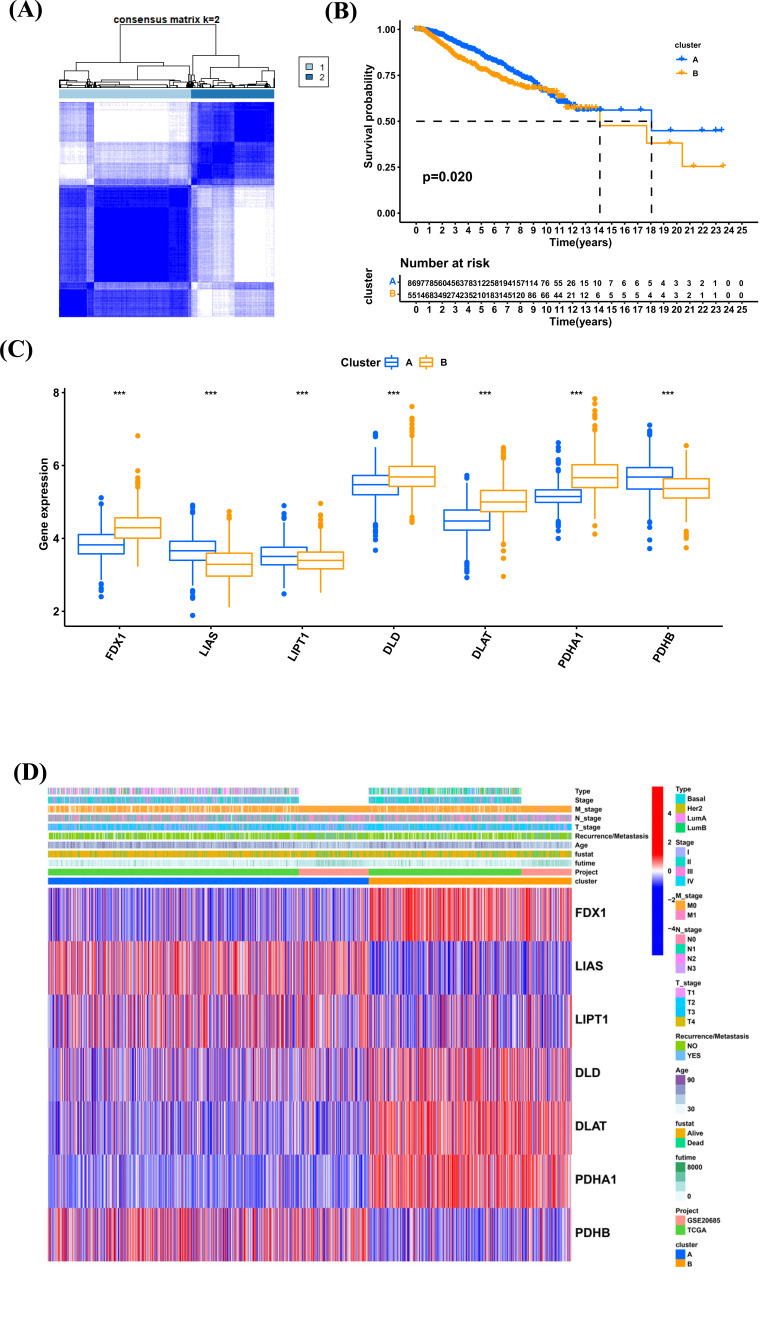
Identification of subtypes based on cuproptosis genes. (**A**) Consensus matrix heatmap defining various clusters and their correlation area. (**B**) Kaplan-Meier analysis of BRCA patients in two clusters. (**C**) The expression of cuproptosis genes in clusters A and B. (**D**). Distributions of clinical features and expression levels of cuproptosis genes between the two clusters. *P < 0.05, **P < 0.01, ***P < 0.001.

Based on the expression of cuproptosis genes, we identified two subtypes (cluster A and cluster B) of BRCA (Fig. **4A**). There was significant survival difference in two clusters (Fig. **4B**). FDX1, DLD, DLAT, and PDHA1 were highly expressed in cluster B, while LIAS, LIPT1, and PDHB were highly expression in cluster A (Fig. **4C**). (Fig. **4D**) displayed the distribution of clinical features and expression of cuproptosis genes in two clusters.

**Fig. (5):**
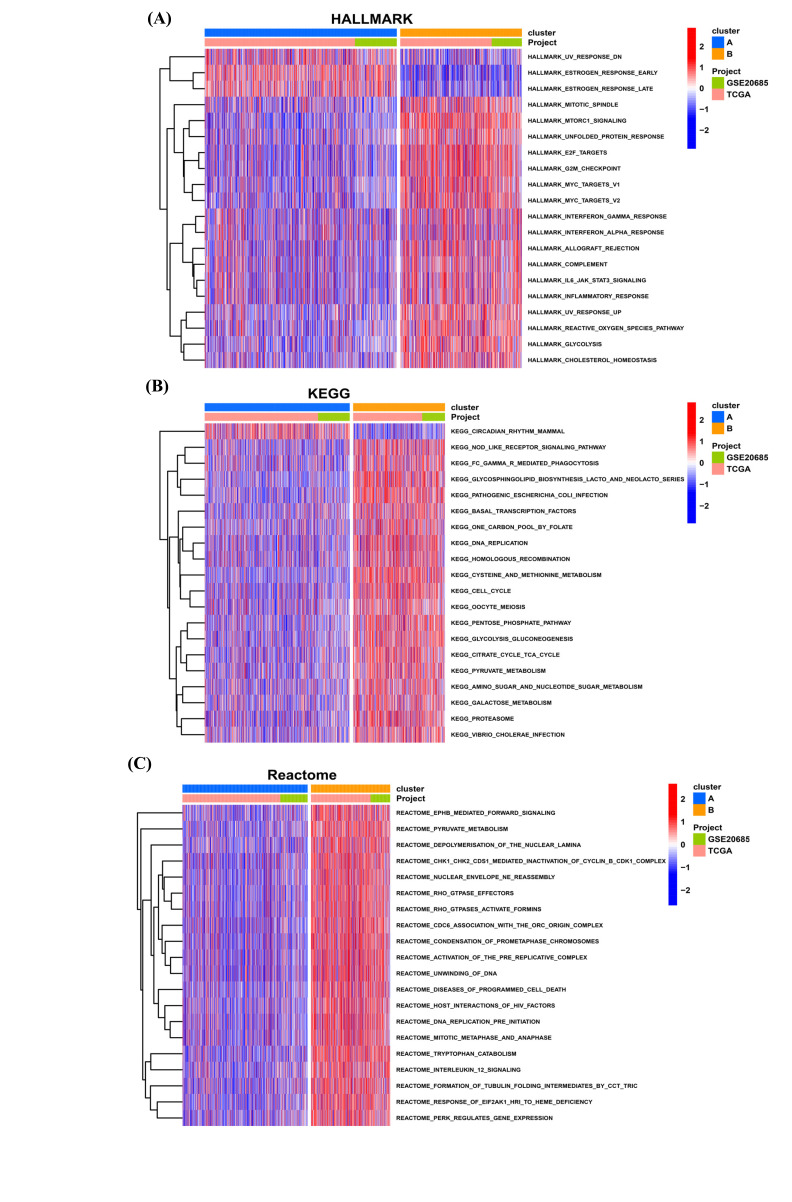
The difference between the two subtypes. (**A**) The score of HALLMARK pathways in two clusters. (**B**) The score of KEGG pathways in two clusters. (**C**) The score of Reactome pathways in two clusters.

We further conducted GSVA to reveal the difference between two clusters. For HALLMARK pathways, the scores of immune-related pathways, including interferon_gamma_response, interferon_alpha_response, and inflammatory_response pathways, were higher in cluster B, indicating a relative immune-activation microenvironment (Fig. **5A**). For KEGG, the scores of cell cycle-related pathways were higher in cluster B, such as DNA replication and cell cycle (Fig. **5B**). For Reactome results, immune-related pathways and cell cycle-related pathways were also observed to be higher in cluster B (Fig. **5C**). These results suggested that the biggest difference between the two subtypes lies in the change in immune microenvironment.

**Fig. (6):**
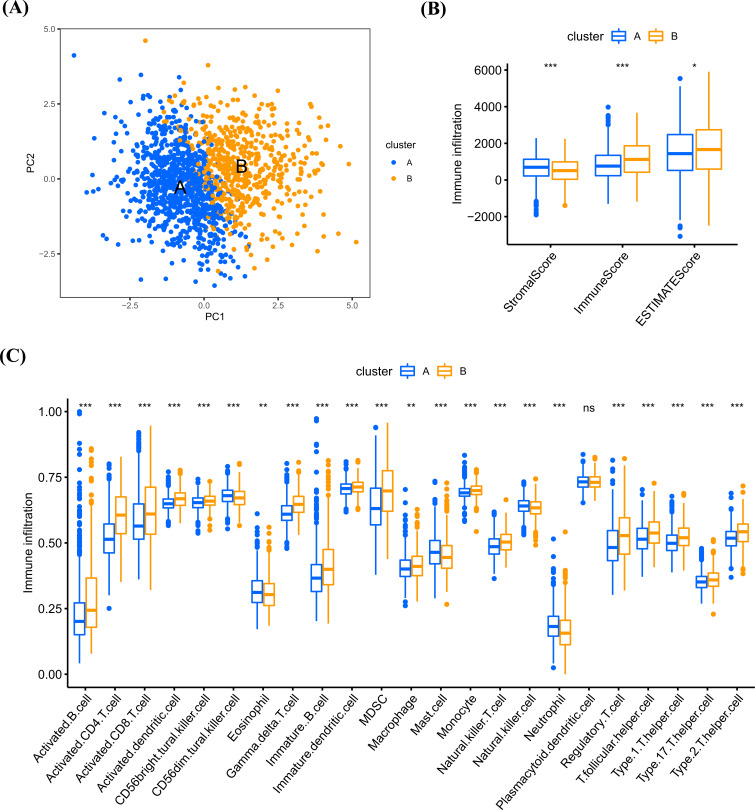
Immune cell infiltration analysis. (**A**) PCA plot displayed the distribution of BRCA samples in two clusters. (**B**) The tumor microenvironment difference between two clusters. (**C**) The difference of immune cell infiltration level in two clusters. *P < 0.05, **P < 0.01, ***P < 0.001.

By performing PCA analysis, we found a clear distinction between clusters A and B (Fig. **6A**). We further estimated the immune infiltration information of BRCA samples. The stromalscore, immunescore, and ESTIMATEscore were all higher in cluster B (Fig. **6B**). The immune cell levels were highly infiltrated in cluster B, except Eosinophil, Mast cell, Neutrophil, and Plasmacytoid dendritic cell (Fig. **6C**).

### 3.3 Identification of Gene Subtypes Based on DEGs

**Fig. (7):**
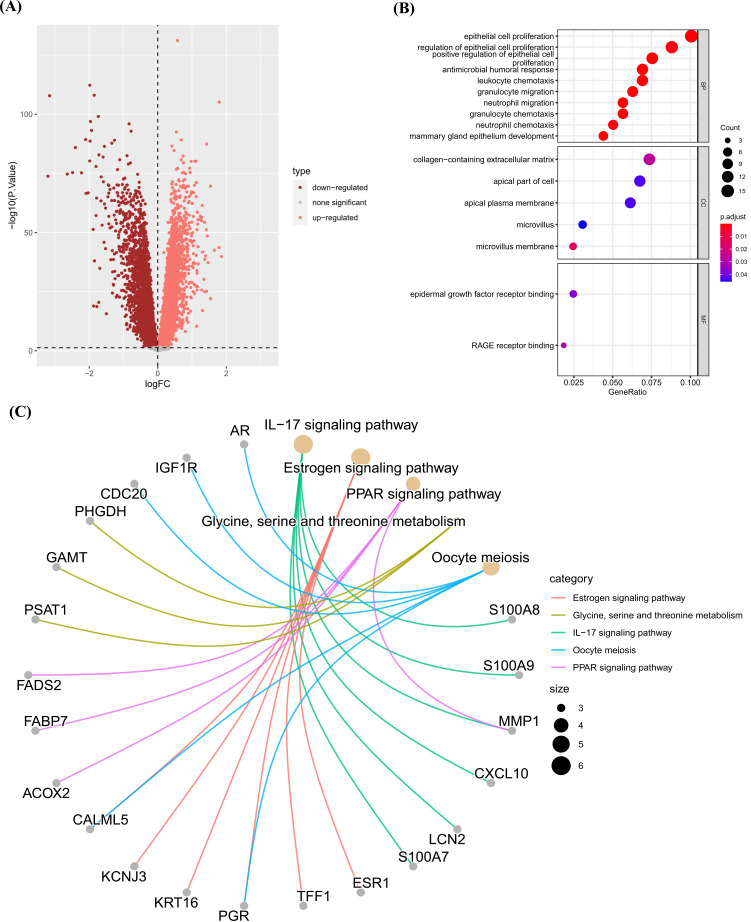
Enrichment analysis. (**A**) The volcano map of DEGs between two clusters. (**B**) GO enrichment analysis of DEGs, including BP, CC, and MF. (**C**) Top5 terms of KEGG results.

We screened 174 DEGs between two subtypes using “limma” package (Fig. **7A**, Supplementary Table **1**). Enrichment analysis of GO revealed that these DEGs were enriched in epithelial cell proliferation, leukocyte chemotaxis, neutrophil migration, and collagen−containing extracellular matrix (Fig. **7B**). For KEGG, DEGs were enriched in IL−17 signaling pathway, Estrogen signaling pathway, PPAR signaling pathway, Glycine, serine and threonine metabolism, and Oocyte meiosis (Fig. **7C**).

**Fig. (8):**
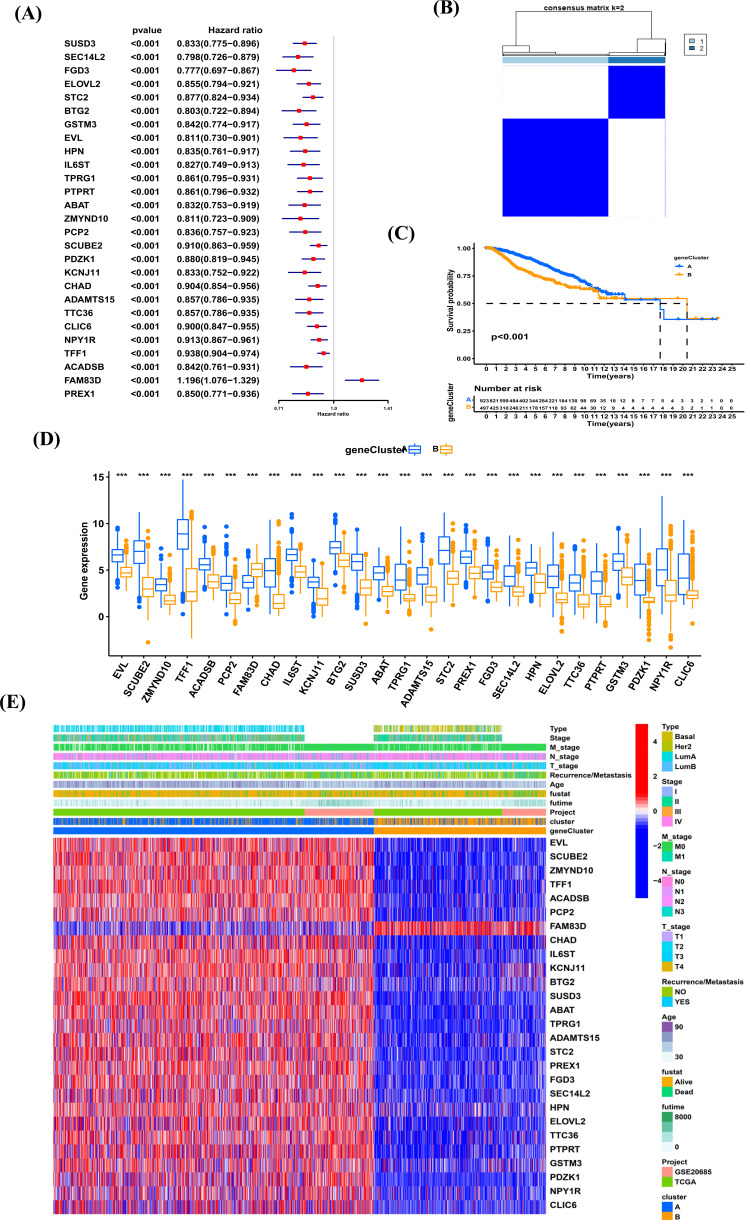
(**A**) The forest map of univariate regression analysis. (**B**) Consensus matrix heatmap defining various clusters and their correlation area. (**C**) Kaplan-Meier analysis of BRCA patients in two geneClusters. (**D**) The expression of 27 genes in two geneClusters. (**E**). Distributions of clinical features and expression levels of 27 DEGs between the two geneClusters. *P < 0.05, **P < 0.01, ***P < 0.001.

We then performed univariate Cox regression analysis to identify the prognostic value of 174 DEGs and screened out 27 genes related to overall survival (*p <* 0.001) (Fig. **8A**, Supplementary Table **2**). To further validate the above results, unsupervised clustering was performed to divide patients into two subtypes based on 27 prognostic genes; namely, geneCluster A-B (Fig. **8B**). BRCA patients in geneCluster A have better survival than patients in geneCluster B (Fig. **8C**). Figs. (**8D** and **8E**) displayed the distribution of clinical features and expression of 27 prognostic genes in two geneClusters.

### 3.4 Construction of Cuproptosis Score

**Fig. (9):**
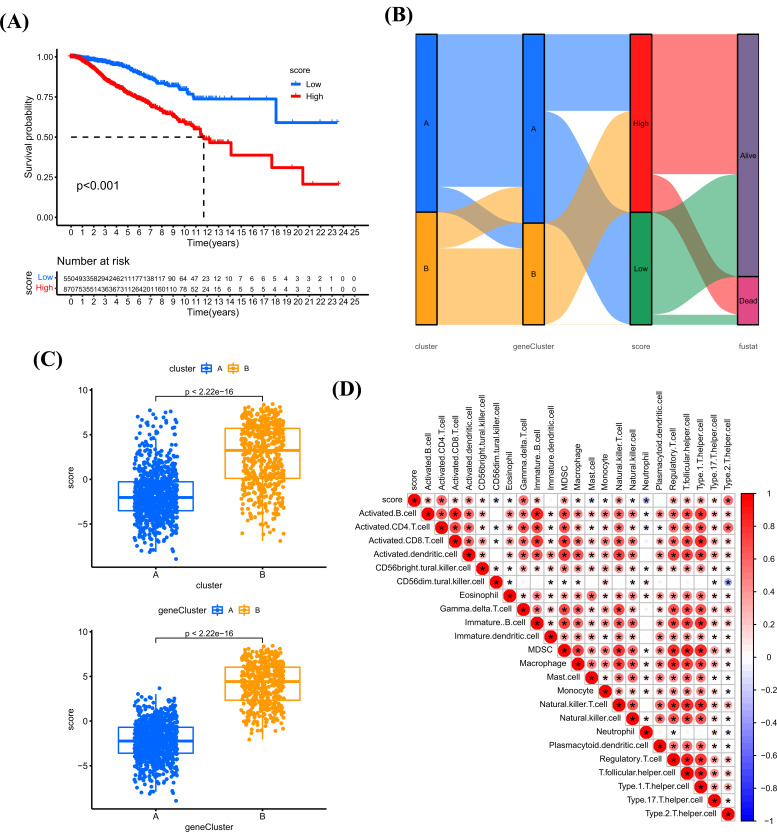
Construction of cuproptosis score. (**A**) Kaplan-Meier analysis of cuproptosis score in BRCA. (**B**) The sankey diagram visualized the correlation between cluster, geneCluster, cuproptosis score, and survival status of BRCA patients. (**C**) UP: The cuproptosis score in cluster A and cluster B; DOWN: The cuproptosis score in geneCluster A and geneCluster B. (**D**) The correlation between cuproptosis score and immune cell infiltration. Red color represents positive correlation, blue color represents negative correlation. *P < 0.05, **P < 0.01, ***P < 0.001.

The cuproptosis score was established based on the 27 prognostic genes using PCA algorithm. Patients with high cuproptosis score have worse survival status (Fig. **9A**). The Sankey diagram visualized the correlation between cluster, geneCluster, cuproptosis score, and survival status (Fig. **9B**). Combined with cluster and geneCluster mentioned above, we found that patients in cluster B or geneCluster B have higher cuproptosis score (Fig. **9C**). The correlation of cuproptosis score with immune cell infiltration indicated that cuproptosis score was positively associated with the infiltration level of most immune cells (Fig. **9D**).

**Fig. (10):**
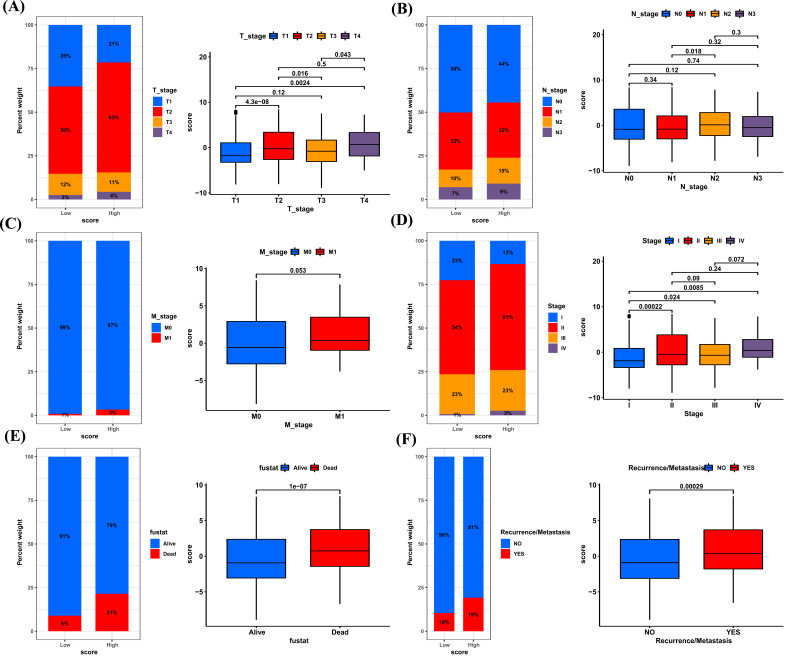
The association of cuproptosis score with clinical features. (**A-F**) The percentage of BRCA patients with various T stages (**A**), N stages (**B**), M stages (**C**), stages (**D**), survival status (**E**), and recurrence or metastasis (**F**) in high and low cuproptosis score groups. *P < 0.05, **P < 0.01, ***P < 0.001.

In addition, we analyzed the association of cuproptosis score with clinical features in BRCA. For T stage, the cuproptosis score was highest in T4 stage (Fig. **10A**). For N stage, the cuproptosis score was highest in N3 stage (Fig. **10B**). For M stage, there was no significant difference between M1 and M0 stages (Fig. **10C**). For stages, the cuproptosis score was highest in stage IV (Fig. **10D**). The percentage of living patients in the high cuproptosis score group (79%) was lower than that in low cuproptosis score group (91%) (Fig. **10E**). Dead patients have higher cuproptosis score than alive patients. Additionally, cuproptosis score was higher in patients with recurrence or metastasis (Fig. **10F**). These results indicated that a high cuproptosis score was generally associated with worse clinical features.

### 3.5 The Role of Cuproptosis Score in Predicting Efficiency of ICI Treatment

**Fig. (11):**
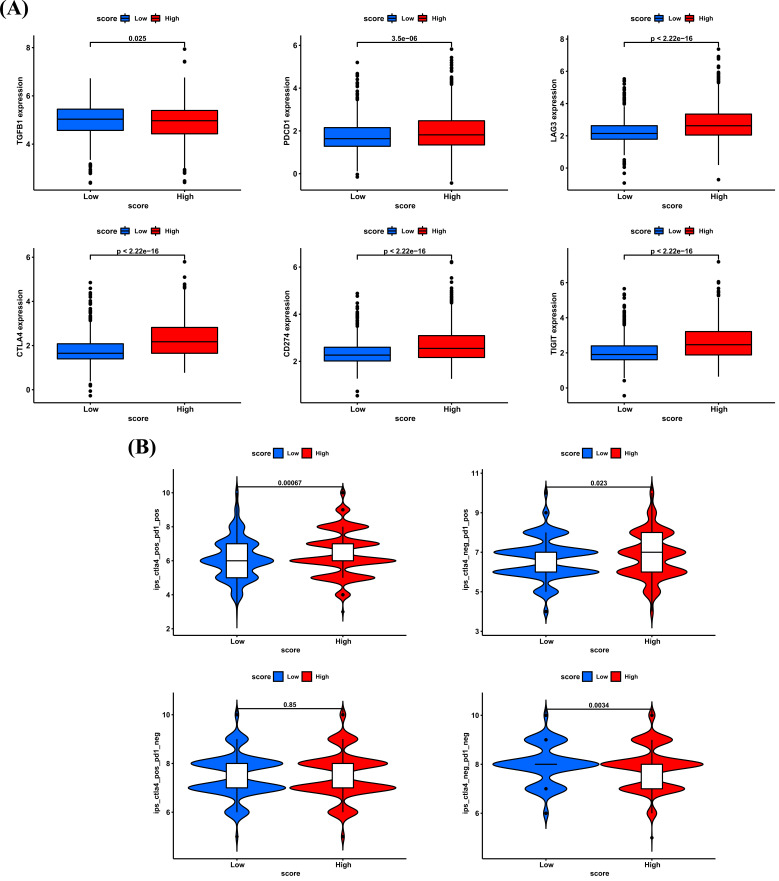
The role of cuproptosis score in predicting efficiency of ICI treatment. (**A**) The indicated immune checkpoints expression in high and low cuproptosis score groups. (**B**) The indicated IPS score of BRCA patients in high and low cuproptosis score groups. *P < 0.05, **P < 0.01, ***P < 0.001.

**Fig. (12):**
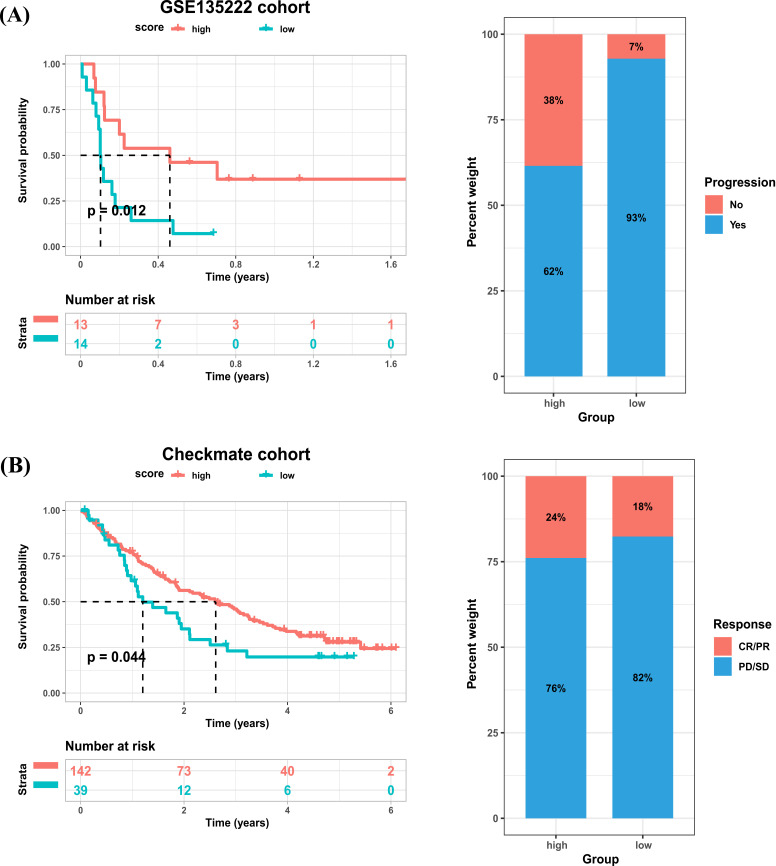
The validation of cuproptosis score in predicting efficiency of ICI treatment. (**A**) LEFT: Kaplan-Meier analysis of patients in high and low cuproptosis score groups in GSE135222 cohort. RIGHT: The percentage of patients with different progress status after ICI treatment in high and low cuproptosis score groups in GSE135222 cohort. (**B**) LEFT: Kaplan-Meier analysis of patients in high and low cuproptosis score groups in Checkmate cohort. RIGHT: The percentage of patients with different progress status after ICI treatment in high and low cuproptosis score groups in Checkmate cohort.

Immune checkpoint is one of the predictors of the efficacy of ICI treatment [4, 5]. The higher the expression of immune checkpoint, the better the efficacy of ICI treatment [6, 7]. We found that common immune checkpoints, such as PDCD1, LAG3, CTLA4, CD274 and TIGIT were highly expressed in high cuproptosis score group (Fig. **11A**). It was reported that patients with high expression of TGFB1 have immunosuppressive microenvironment and are resistant to ICI treatment [8-11]. We found that TGFB1 expression was lower in high cuproptosis score group (Fig. **11A**). These results suggested that patients with high score may be sensitive to ICI treatment. As there is no publicly available immunotherapy dataset in BRCA, we used the immunophenoscore (IPS) calculated by TCIA database to predict the efficiency of ICI treatment. Results indicated that patients in high cuproptosis score group were more sensitive to ICI treatment (Fig. **11B**). Moreover, by analyzing the immunotherapy datasets, we proved that patients with high cuproptosis scores were sensitive to ICI treatment in GSE135222 cohort (Fig. **12A**, Advanced non-small cell lung carcinoma, anti-PD-1/PD-L1) and checkmate cohort (Fig. **12B**, Kidney renal clear cell carcinoma, anti-PD-1). These results indicated that cuproptosis score may contribute to the prediction of the efficiency of ICI treatment in BRCA.

**Fig. (13):**
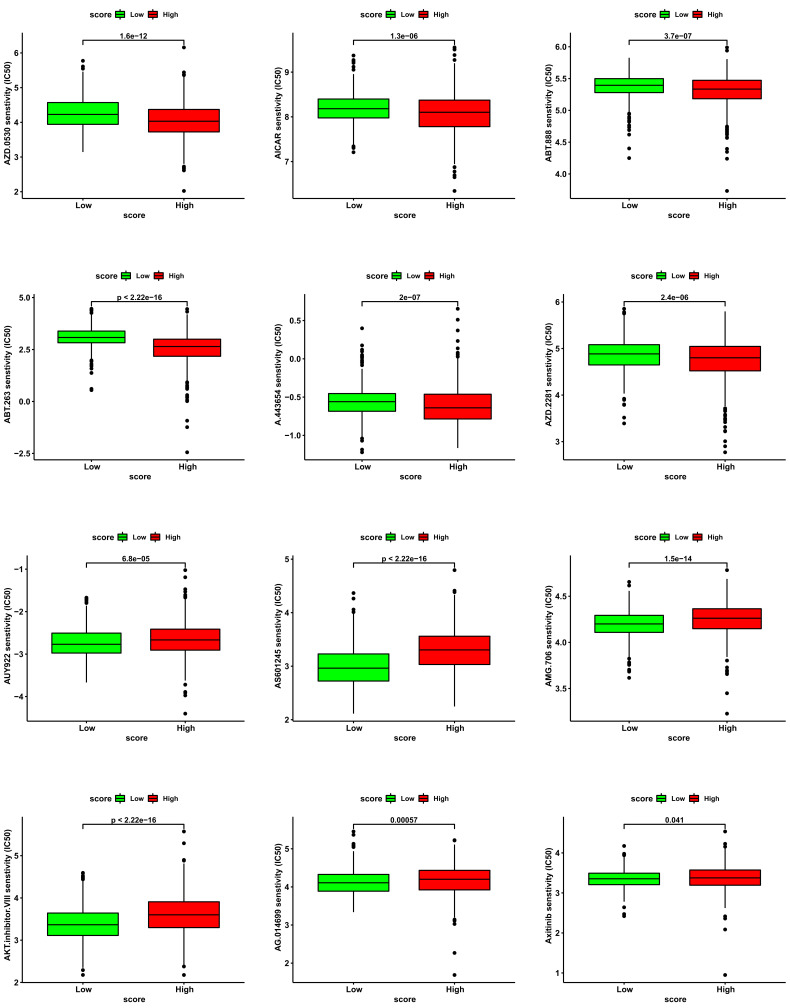
The analysis of curative effect of anti-tumor drugs.(**A-B**) The IC50 of indicated anti-tumor drugs in high and low cuproptosis score groups. *P < 0.05, **P < 0.01, ***P < 0.001.

In addition to immunotherapy, we also analyzed conventional anti-tumor drugs [12]. Based on the predicted results by R package “pRRophetic”, we displayed 6 anti-tumor drugs that may be resistant to patients with high cuproptosis score (Fig. **13A**) and 6 anti-tumor drugs that may be sensitive to patients with high cuproptosis score (Fig. **13B**).

## 5 DISCUSSION

Copper is a double-edged sword, which is an essential enzyme cofactor [13, 14]. However, even if the concentration of copper ions in cells is not high, it may be toxic and lead to cell death [15-17]. Recent studies have revealed that unbalanced copper homeostasis could affect tumor growth, causing irreversible damage [18-20]. Copper can induce multiple forms of cell death, including apoptosis and autophagy, through various mechanisms, including reactive oxygen species accumulation, proteasome inhibition, and antiangiogenesis [21-24]. The latest research shows that copper-induced death (Cuproptosis) is a new cell death mechanism, which is distinct from all other known mechanisms of regulated cell death, including apoptosis, ferroptosis, pyroptosis, and necroptosis [2, 25]. However, the role of cuproptosis in tumor development and progression remains unclear.

In our study, a total of 7 cuproptosis genes were enrolled, including FDX1, LIAS, LIPT1, DLD, DLAT, PDHA1, and PDHB. We explored CNV and SNV of cuproptosis genes. Among them, DLD has the highest amplification frequency in BRCA. SNV results indicated that the mutation frequency of cuproptosis genes was low in BRCA. PDHB was highly expressed in BRCA, while DLD, FDX1, PDHA1, LIPT1, and LIAS were lowly expressed in BRCA.

Based on the expression of 7 cuproptosis genes, we identified two subtypes (cluster A and cluster B) of BRCA. Compared to patients with cluster A, patients with cluster B had worse clinical features and bad survival. We further conducted GSVA to reveal the difference between two subtypes. The results of HALLMARK, Reactome, and KEGG all revealed an enrichment of immune-related pathways in cluster B, such as interferon_gamma_response and interferon_alpha_response. We further estimated the infiltration level of 23 immune cells and found that 18 of 23 immune cells were highly infiltrated in cluster B.

We further screened 174 DEGs between two subtypes. Enrichment analysis revealed that these DEGs were enriched in immune-related pathways, such as IL-17 signaling pathway. We then performed univariate Cox regression analysis to identify the prognostic value of 174 DEGs and screened out 27 genes related to overall survival (*p <* 0.001). The cuproptosis score was established based on the 27 prognostic genes using PCA algorithm. Patients with high cuproptosis score have worse survival status. The correlation of cuproptosis score with immune cell infiltration indicated that cuproptosis score was positively associated with immune cell infiltration level. By analyzing the association of cuproptosis score with clinical features in BRCA. We found that high cuproptosis score was generally associated with worse clinical features, such as higher TNM stage, dead status, and recurrence or metastasis.

Immune checkpoint is one of the predictors of the efficacy of ICI treatment [5, 21]. The higher the expression of immune checkpoint, the better the efficacy of ICI treatment [5, 21]. We found that common immune checkpoints, such as PDCD1, LAG3, CTLA4, CD274 and TIGIT were highly expressed in high cuproptosis score group. It was reported that patients with high expression of TGFB1 have immunosuppressive microenvironment and are resistant to ICI treatment [8-10]. We found that TGFB1 expression was lower in high cuproptosis score group. These results suggested that patients with high score may be sensitive to ICI treatment. As there is no publicly available immunotherapy dataset in BRCA, we used the immunophenoscore (IPS) calculated by TCIA database to predict the efficiency of ICI treatment. Results indicated that patients in high cuproptosis score group were more sensitive to ICI treatment. Moreover, by analyzing the immunotherapy datasets, we proved that patients with high cuproptosis score were sensitive to ICI treatment in GSE135222 cohort and checkmate cohort. These results indicated that cuproptosis score may contribute to the prediction of the efficiency of ICI treatment in BRCA.

## CONCLUSION

We conducted a comprehensive analysis of cuproptosis genes and revealed its extensive regulatory mechanisms affecting tumor immune microenvironment, clinical features and prognosis of BRCA patients. We also constructed a cuproptosis score and determined its reliability in predicting prognosis and ICI treatment efficacy. These findings highlight the important clinical significance of cuproptosis genes and provide a new idea for guiding the personalized immunotherapy strategy of BRCA patients.

## Data Availability

The data used and analyzed during the current study are available from the principal author, Jialin Li, on reasonable request.

## LIST OF ABBREVIATIONS

BRCA: Breast Carcinoma
DEGs: Differentially Expressed Genes
TCGA: The Cancer Genome Atlas
GO: Gene Ontology
KEGG: Kyoto Encyclopedia of Genes and Genomes
ssGSEA: Single-sample Gene Set Enrichment Analysis
PCA: Principal Component Analysis
SNV: Single Nucleotide Variants
CNV: Copy Number Variant
IPS: Immunophenoscore
GSVA: Gene Set Variation Analysis
ICI: Immune Checkpoint Inhibitor

## References

[1] Kahlson M.A., Dixon S.J. (2022). Copper-induced cell death.. Science.

[2] Tsvetkov P., Coy S., Petrova B., Dreishpoon M., Verma A., Abdusamad M., Rossen J., Joesch-Cohen L., Humeidi R., Spangler R.D., Eaton J.K., Frenkel E., Kocak M., Corsello S.M., Lutsenko S., Kanarek N., Santagata S., Golub T.R. (2022). Copper induces cell death by targeting lipoylated TCA cycle proteins.. Science.

[3] Yang Z., Ming X., Huang S., Yang M., Zhou X., Fang J. (2021). Comprehensive analysis of m6A regulators characterized by the immune cell infiltration in head and neck squamous cell carcinoma to aid immunotherapy and chemotherapy.. Front. Oncol..

[4] Bagchi S., Yuan R., Engleman E.G. (2021). Immune checkpoint inhibitors for the treatment of cancer: Clinical impact and mechanisms of response and resistance.. Annu. Rev. Pathol..

[5] Pinato D.J., Howlett S., Ottaviani D., Urus H., Patel A., Mineo T., Brock C., Power D., Hatcher O., Falconer A., Ingle M., Brown A., Gujral D., Partridge S., Sarwar N., Gonzalez M., Bendle M., Lewanski C., Newsom-Davis T., Allara E., Bower M. (2019). Association of prior antibiotic treatment with survival and response to immune checkpoint inhibitor therapy in patients with cancer.. JAMA Oncol..

[6] Liu D., Lin J.R., Robitschek E.J., Kasumova G.G., Heyde A., Shi A., Kraya A., Zhang G., Moll T., Frederick D.T., Chen Y.A., Wang S., Schapiro D., Ho L.L., Bi K., Sahu A., Mei S., Miao B., Sharova T., Alvarez-Breckenridge C., Stocking J.H., Kim T., Fadden R., Lawrence D., Hoang M.P., Cahill D.P., Malehmir M., Nowak M.A., Brastianos P.K., Lian C.G., Ruppin E., Izar B., Herlyn M., Van Allen E.M., Nathanson K., Flaherty K.T., Sullivan R.J., Kellis M., Sorger P.K., Boland G.M. (2021). Evolution of delayed resistance to immunotherapy in a melanoma responder.. Nat. Med..

[7] Zhang X., Zhang X., Li G., Hao Y., Liu L., Zhang L., Chen Y., Wu J., Wang X., Yang S., Xu S. (2022). A novel necroptosis-associated lncRNA signature can impact the immune status and predict the outcome of breast cancer.. J. Immunol. Res..

[8] Cuenca-Micó O., Delgado-González E., Anguiano B., Vaca-Paniagua F., Medina-Rivera A., Rodríguez-Dorantes M., Aceves C. (2021). Effects of molecular iodine/chemotherapy in the immune component of breast cancer tumoral microenvironment.. Biomolecules.

[9] Vishnubalaji R., Alajez N.M. (2021). Epigenetic regulation of triple negative breast cancer (TNBC) by TGF-β signaling.. Sci. Rep..

[10] Wang J., Xiang H., Lu Y., Wu T. (2021). Role and clinical significance of TGF β1 and TGF βR1 in malignant tumors (Review).. Int. J. Mol. Med..

[11] Chang L.S., Barroso-Sousa R., Tolaney S.M., Hodi F.S., Kaiser U.B., Min L. (2019). Endocrine toxicity of cancer immunotherapy targeting immune checkpoints.. Endocr. Rev..

[12] Minami H., Kiyota N., Kimbara S., Ando Y., Shimokata T., Ohtsu A., Fuse N., Kuboki Y., Shimizu T., Yamamoto N., Nishio K., Kawakami Y., Nihira S., Sase K., Nonaka T., Takahashi H., Komori Y., Kiyohara K. (2021). Guidelines for clinical evaluation of anti‐cancer drugs.. Cancer Sci..

[13] Harris E.D. (1992). Copper as a cofactor and regulator of copper,zinc superoxide dismutase.. J. Nutr..

[14] Solano F. (2018). On the metal cofactor in the tyrosinase family.. Int. J. Mol. Sci..

[15] Jiang Y., Huo Z., Qi X., Zuo T., Wu Z. (2022). Copper-induced tumor cell death mechanisms and antitumor theragnostic applications of copper complexes.. Nanomedicine (Lond.).

[16] Mufti A.R., Burstein E., Duckett C.S. (2007). XIAP: Cell death regulation meets copper homeostasis.. Arch. Biochem. Biophys..

[17] Han J., Hu Y., Liu S., Jiang J., Wang H. (2022). A newly established cuproptosis-associated long non-coding RNA signature for predicting prognosis and indicating immune microenvironment features in soft tissue sarcoma.. J. Oncol..

[18] De Luca A., Barile A., Arciello M., Rossi L. (2019). Copper homeostasis as target of both consolidated and innovative strategies of anti-tumor therapy.. J. Trace Elem. Med. Biol..

[19] Shanbhag V.C., Gudekar N., Jasmer K., Papageorgiou C., Singh K., Petris M.J. (2021). Copper metabolism as a unique vulnerability in cancer.. Biochim. Biophys. Acta Mol. Cell Res..

[20] Li Y. (2020). Copper homeostasis: Emerging target for cancer treatment.. IUBMB Life.

[21] Liu L., Bai X., Wang J., Tang X.R., Wu D.H., Du S.S., Du X.J., Zhang Y.W., Zhu H.B., Fang Y., Guo Z.Q., Zeng Q., Guo X.J., Liu Z., Dong Z.Y. (2019). Combination of TMB and CNA stratifies prognostic and predictive responses to immunotherapy across metastatic cancer.. Clin. Cancer Res..

[22] Singh N.K., Kumbhar A.A., Pokharel Y.R., Yadav P.N. (2020). Anticancer potency of copper(II) complexes of thiosemicarbazones.. J. Inorg. Biochem..

[23] Fei B.L., Hui C.N., Wei Z., Kong L.Y., Long J.Y., Qiao C., Chen Z.F. (2021). Copper(II) and iron(III) complexes of chiral dehydroabietic acid derived from natural rosin: Metal effect on structure and cytotoxicity.. Metallomics.

[24] Lin L.S., Huang T., Song J., Ou X.Y., Wang Z., Deng H., Tian R., Liu Y., Wang J.F., Liu Y., Yu G., Zhou Z., Wang S., Niu G., Yang H.H., Chen X. (2019). Synthesis of copper peroxide nanodots for H2O2 self-supplying chemodynamic therapy.. J. Am. Chem. Soc..

[25] Song S., Zhang M., Xie P., Wang S., Wang Y. (2022). Comprehensive analysis of cuproptosis-related genes and tumor microenvironment infiltration characterization in breast cancer.. Front. Immunol..

